# Salvage chemoradiotherapy with cisplatin and vinorelbine for postoperative locoregional recurrence of non-small cell lung cancer

**DOI:** 10.1097/MD.0000000000008635

**Published:** 2017-11-27

**Authors:** Kakeru Hisakane, Kiyotaka Yoh, Naoki Nakamura, Hibiki Udagawa, Keisuke Kirita, Shigeki Umemura, Shingo Matsumoto, Seiji Niho, Tetsuo Akimoto, Masahiro Tsuboi, Koichi Goto

**Affiliations:** a Department of Thoracic Oncology; b Department of Radiation Oncology; c Department of Thoracic Surgery, National Cancer Center, Kashiwa, Japan.

**Keywords:** Cisplatin and vinorelbine, locoregional recurrence, non-small cell lung cancer, salvage chemoradiotherapy

## Abstract

Although a few investigators have demonstrated the effect of concurrent chemoradiotherapy (CRT) for postoperative recurrent non-small cell lung cancer (NSCLC), the outcome of this treatment remains unclear. The aim of this study was to elucidate the efficacy and tolerability of concurrent CRT with cisplatin (CDDP) and vinorelbine (VNR) in patients with postoperative locoregional recurrent NSCLC. A total of 40 patients who had received concurrent CRT with CDDP and VNR between January 1999 and December 2014 were retrospectively analyzed. Patients were treated with CDDP (80 mg/m^2^ on day 1) and VNR (20 mg/m^2^ on days 1 and 8) every 4 weeks. Radiotherapy was administered concurrently during cycle 1. The delivered x-ray radiation dose was 60 Gy in all 37 patients who received x-ray radiotherapy; 3 patients received proton beam radiation (66 Gy [RBE] in 1 patient and 60 Gy [RBE] in 2 patients). The objective response rate was 85% (95% confidence interval [CI], 70.9%–92.9%). The median progression-free survival was 20.3 months (95% CI, 12.9 months–not reached). The 2-year survival rate was 78.9% (95% CI, 63.0%–89.1%). The most common grade ≥3 toxicity was neutropenia (18%). No grade ≥3 radiation pneumonitis and no treatment-related deaths were observed.

Our study revealed that concurrent CRT with CDDP and VNR was active and safe for patients with postoperative locoregional recurrent NSCLC. Salvage CRT for postoperative locoregional recurrent NSCLC might be a promising treatment for selected patients.

## 1 Introduction

Lung cancer is the main cause of cancer-related mortality worldwide. Even after the complete resection of non-small cell lung cancer (NSCLC), postoperative recurrences occur in approximately 30% to 40% of patients.^[1–5]^ The types of postoperative recurrences are usually categorized into distant recurrence, locoregional recurrence, and combined recurrence.^[6]^ The majority of cases of recurrent NSCLC after surgery involve distant metastasis with or without locoregional recurrence.^[6]^ In such patients, the administration of systemic chemotherapy is considered to be the treatment of choice based on evidence for the treatment of original stage IV disease.^[6]^ Meanwhile, the incidence of postoperative locoregional recurrent NSCLC without distant metastasis among all postoperative recurrent NSCLC ranges from 5% to 30%.^[1–3,7–12]^ In clinical practice, local salvage therapy, such as surgical resection and radiotherapy with or without chemotherapy, are usually considered for the treatment of such patients with postoperative locoregional recurrent disease.

To date, several retrospective studies evaluating the efficacy and safety of radiotherapy with or without chemotherapy for patients with postoperative locoregional recurrent NSCLC have been published.^[7,8,11–23]^ Some of these investigators have demonstrated that concurrent chemoradiotherapy (CRT) improved survival compared with radiotherapy alone.^[20,23]^ Recently, promising outcomes of concurrent CRT for recurrent NSCLC have been reported.^[12,22]^ However, whether the results of concurrent CRT for postoperative locoregional recurrent disease are equal to those for de novo stage III disease remains unclear, as the selected chemotherapeutic regimens and the delivered radiation doses have varied for each patient in previous studies.

In this context, we conducted a retrospective analysis of concurrent CRT with cisplatin (CDDP) and vinorelbine (VNR) to evaluate the efficacy and tolerability of this treatment in patients with postoperative locoregional recurrent NSCLC.

## 2 Material and methods

### 2.1 Patient selection

**Figure 1:**
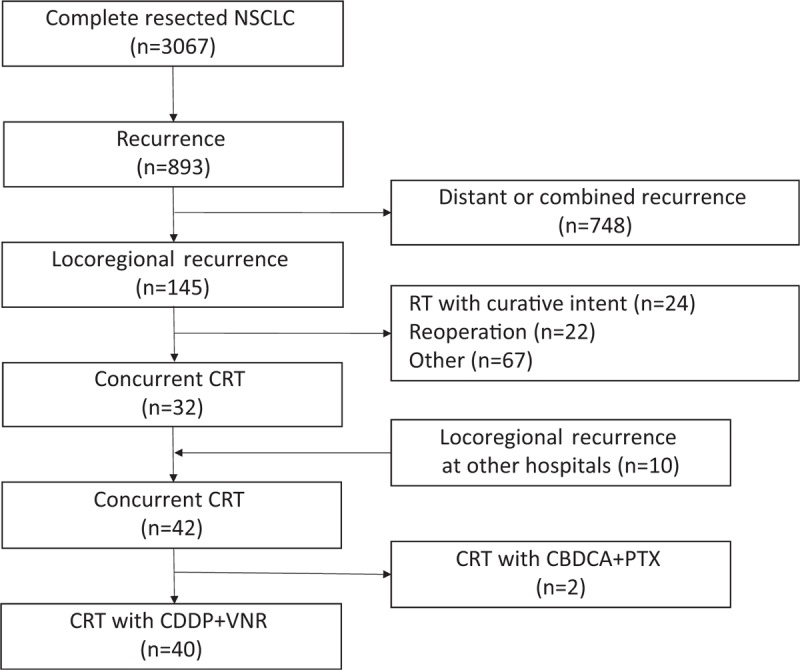
Patient disposition. CBDCA = carboplatin, CDDP = cisplatin, CRT = chemoradiotherapy, NSCLC = non-small cell lung cancer, PTX = paclitaxel, RT = radiotherapy, VNR = vinorelbine.

Between January 1999 and December 2013, a total of 3067 consecutive patients with NSCLC underwent a complete resection at our institute. The patient flow diagram is shown in Figure 1. As of the end of 2014, a total of 893 patients (29%) had been diagnosed as having recurrent disease. Among these 893 patients, 145 patients (16%) had a locoregional recurrence without distant metastases. Thirty-two of these patients received concurrent CRT as a local therapy with curative intent. Furthermore, 10 patients who had undergone a curative resection at another institution and who had been identified as having locoregional recurrent NSCLC also received concurrent CRT at our hospital between January 1999 and December 2014. Among these 42 patients, 40 patients received concurrent CRT with CDDP and VNR, and these patients were retrospectively analyzed in the present study. All the clinical data were retrieved from the patients’ medical records.

In this study, locoregional recurrence was defined as a recurrence at one of the following sites: ipsilateral hilar lymph node, ipsilateral mediastinum lymph node, ipsilateral supraclavicular lymph node, contralateral mediastinum lymph node, contralateral supraclavicular lymph node, or an ipsilateral single pulmonary lesion including the surgical margin. Ipsilateral multiple pulmonary lesions, ipsilateral pleural effusion, contralateral hilar lymph nodes, and contralateral pulmonary lesions were regarded as distant metastases. The disease distribution of the locoregional recurrence defined in this study was determined by referring to that of the patients with locally advanced stage III NSCLC for whom definitive radiotherapy could be administered. The treatment policy for all the patients was thoroughly discussed at a joint meeting of several experts on thoracic oncology, radiation oncology, and thoracic surgery. All the patients provided written informed consent before the treatment. The study was conducted with the approval of the Institutional Review Board of the National Cancer Center. The IRB approval number for this study was 2016-309.

### 2.2 Treatment method

The chemotherapy consisted of CDDP (80 mg/m^2^ on day 1) and VNR (20 mg/m^2^ on days 1 and 8). The treatment cycles were repeated every 4 weeks for a maximum of 4 cycles. CDDP and VNR were administered by intravenous infusion. Dose reduction, omission, and the discontinuation of chemotherapy were performed at the physician's discretion.

Radiotherapy was administered concurrently during cycle 1. The clinical target volume (CTV) was equal to the gross tumor volume, and the planning target volume was determined using a 1.0- to 2.0-cm margin from the CTV. Elective nodal irradiation was not administered in principle. Radiotherapy was administered using a 6- to 10-MV x-ray from a linear accelerator using a 3-dimensional conformal technique in 37 patients. All these patients received 60 Gy in 30 fractions (5 fractions per week). Proton beam therapy was delivered using a passive technique in 3 patients. One patient received 66 Gy (RBE) in 33 fractions; 2 patients received 60 Gy (RBE) in 30 fractions.

### 2.3 Evaluation of efficacy and toxicity

The best objective response was evaluated using chest CT according to the Response Evaluation Criteria in Solid Tumors (RECIST) version 1.1. The objective response rate (ORR) was calculated as the total percentage of patients with a complete response (CR) or a partial response (PR). The CT images were independently reviewed by at least 2 pulmonologists.

Toxicity was graded according to the Common Terminology Criteria for Adverse Events, version 4.0.

### 2.4 Statistical analyses

Progression-free survival (PFS) was defined as the time from the date of the initiation of concurrent CRT until the date of disease progression, death from any cause, or the last follow-up. Overall survival (OS) was defined as the time from the date of initiation of concurrent CRT until the date of death from any cause, or the last follow-up. The survival curves were estimated using a Kaplan-Meier analysis; differences in survival were compared using the log-rank test. A multivariate regression analysis was conducted according to the Cox proportional hazard model. Statistical differences were considered significant if *P* < .05. All the statistical analyses were performed using the JMP statistical software package for Windows, version 11 (SAS Institute, Cary, NC).

## 3 Results

### 3.1 Patient characteristics

The characteristics of the 40 patients who received concurrent CRT with CDDP and VNR are summarized in Table 1. The median age at the initiation of concurrent CRT was 64 years (range, 43–76 years), 28 (70%) of the patients were male, 32 (80%) were smokers, and most of the patients (93%) had an Eastern Cooperative Oncology Group Performance Status (PS) of 0. The histological classifications were adenocarcinoma in 31 (78%) patients, squamous cell carcinoma in 8 (20%) patients, and large cell carcinoma in 1 (2%) patients. The pathological stage at surgical resection according to the TNM 7th edition was stage I in 14 (35%) patients, stage II in 9 (22%) patients, and stage III in 17 (43%) patients. As the initial operation, 37 of the 40 (93%) patients underwent a lobectomy, and 3 patients underwent a pneumonectomy. Adjuvant chemotherapy was administered to 14 (35%) patients. The median recurrence-free interval after the initial operation was 18.8 months (range, 2.6–67.1 months).

**Table 1:**
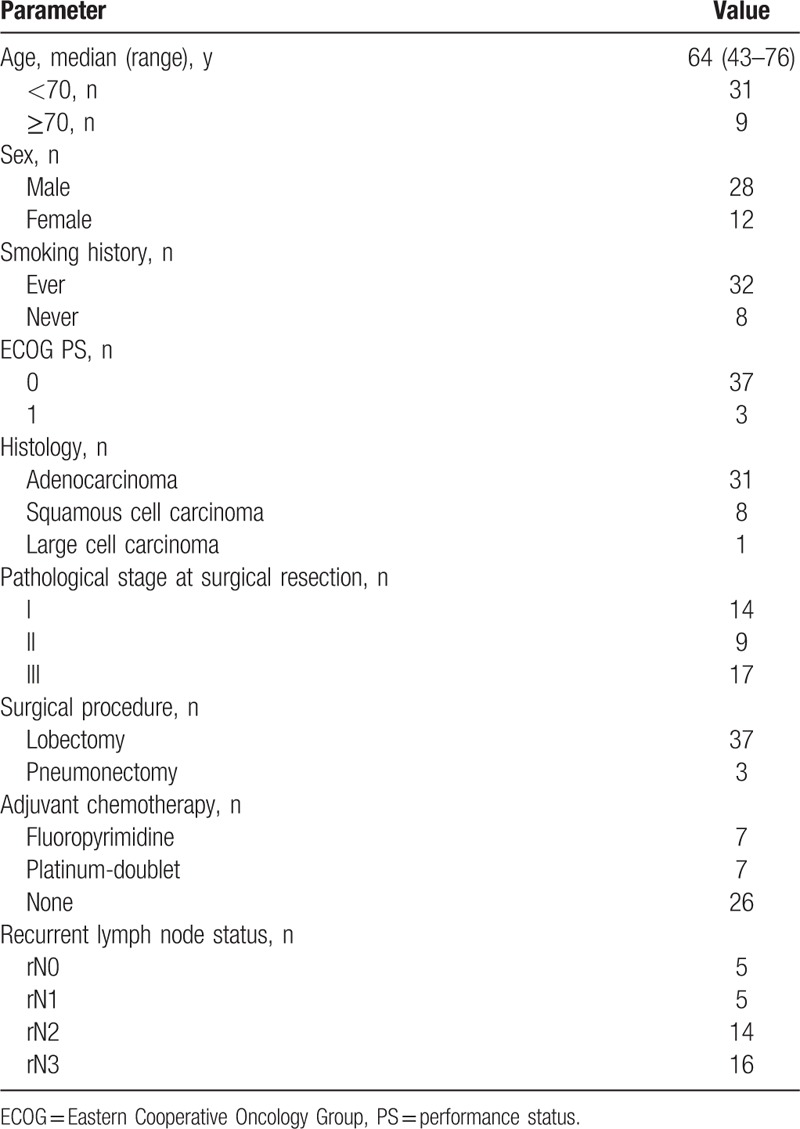
Patient characteristics (N = 40).

The locoregional recurrent sites were divided into lymph node and ipsilateral pulmonary field. Thirty-one (78%) patients had only lymph node recurrence, 5 (12%) patients had only ipsilateral pulmonary field recurrence and 4 (10%) patients had both lymph node and ipsilateral pulmonary field recurrences. Of the 35 patients with lymph node recurrence, 5 (14%) patients had TNM 7^th^ stage rN1, 14 (40%) had stage rN2, and 16 (46%) had stage rN3. As for the number of recurrence sites, 22 (55%) patients had a single recurrence and 18(45%) patients had multiple recurrences. Twenty-seven (68%) patients underwent PET at the time of recurrence. Histological confirmation of recurrent disease was performed for 12 (30%) patients.

### 3.2 Treatment efficacy and survival

Table 2 summarizes the treatment outcome of concurrent CRT with CDDP and VNR. The best objective response of the patients was as follows: 4 had a CR, 30 had a PR, 5 had stable disease, and 1 had progressive disease, yielding an ORR of 85% (95% confidence interval [CI], 70.9%-92.9%). The median number of applied chemotherapy cycles was 3 (range, 2–4 cycles).

**Table 2:**
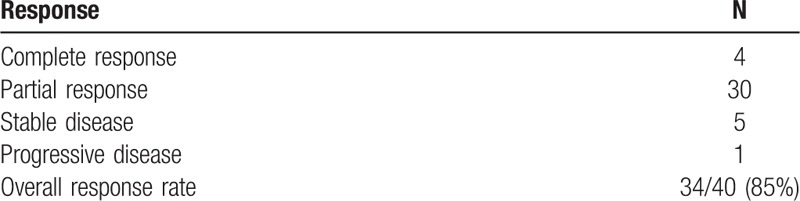
Response to chemoradiotherapy.

**Figure 2:**
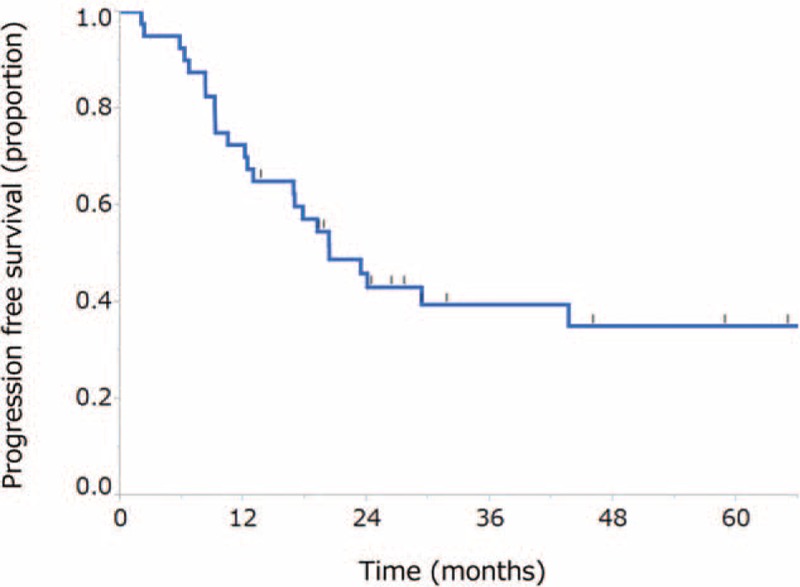
Kaplan-Meier curve for progression-free survival. The median progression-free survival was 20.7 months (95% confidence interval, 12.9 months–not reached).

**Figure 3:**
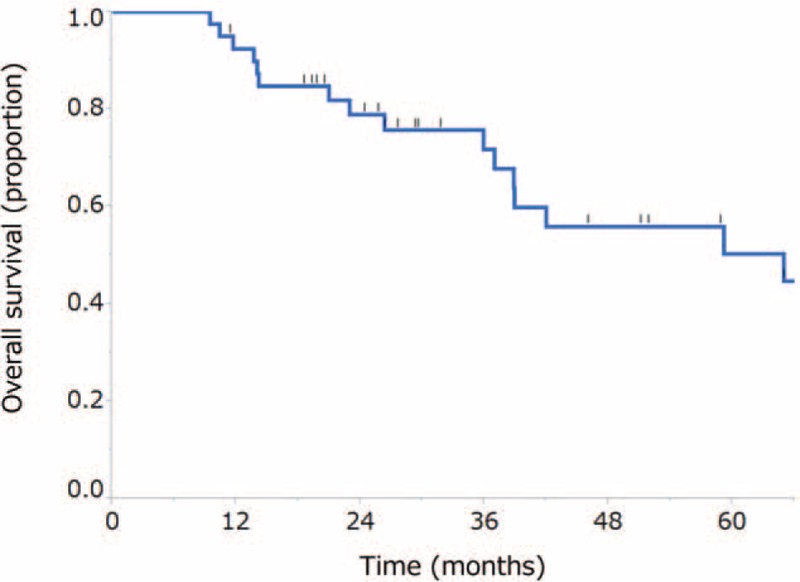
Kaplan-Meier curve for overall survival. The median survival time was 65.0 months (95% confidence interval, 37.0 months–not reached), and the 2-year survival rate was 78.9% (95% confidence interval, 63.0%–89.1%).

The median follow-up time for the surviving patients was 30.7 months (range, 11.4–126 months). The median PFS was 20.3 months (95% CI, 12.9 months-not reached) (Fig. 2). The median OS was 65.0 months (95% CI, 37.0 months–not reached), and the 2-year survival rate was 78.9% (95% CI, 63.0%-89.1%) (Fig. 3). A total of 24 patients (60%) had experienced disease relapse and 18 patients (45%) had died at the time of data cut-off.

A univariate analysis showed that sex significantly influenced the OS (*P* = .02). In a multivariate analysis, a female sex was also identified as an independent predictor of a poor prognosis (*P* = .03) (Table 3).

**Table 3:**
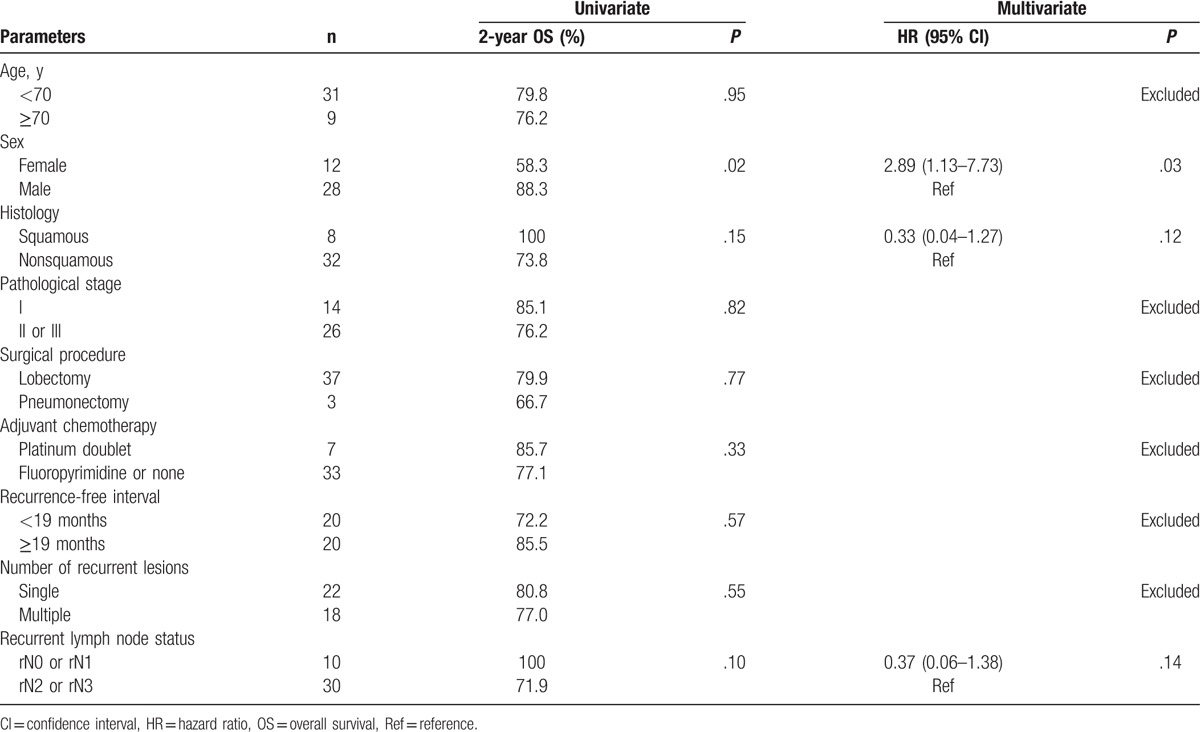
Univariate and multivariate analyses of risk factors for overall survival.

Regarding the initial patterns of recurrence after CRT, only in-field recurrence, both in-field and out-field recurrence, and only out-field recurrence were observed in 3, 4, and 17 patients, respectively. Seventeen patients received systemic chemotherapies after disease relapse.

### 3.3 Toxicity

The toxicities of the concurrent CRT are summarized in Table 4. The most common hematological grade ≥3 adverse event was neutropenia (18%). There were no patients with Grade ≥3 anemia or thrombocytopenia. Esophagitis related to radiotherapy was the most common nonhematological grade ≥3 adverse event, occurring in 5% of the patients. No cases of grade ≥3 radiation pneumonitis were observed, whereas 3 (7%) patients experienced grade 2 radiation pneumonitis. No treatment-related deaths occurred.

**Table 4:**
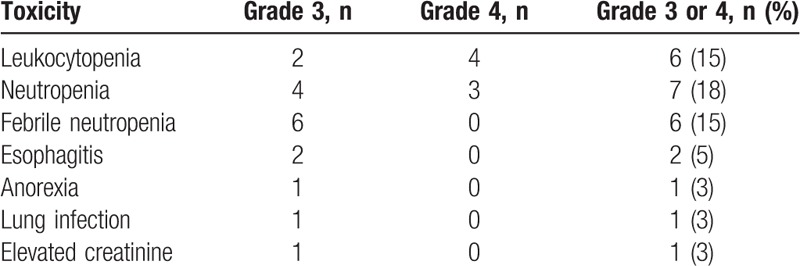
Adverse events.

## 4 Discussion

In the current study, we demonstrated that combination chemotherapy with CDDP, VNR, and simultaneous radiotherapy was effective and safe.

The results of the present study were encouraging, demonstrating an ORR of 85%, a median PFS of 20.7 months, and a 2-year survival rate of 78.9%. Concurrent CRT is considered the standard of care for locally advanced NSCLC.^[24]^ Although the optimal chemotherapeutic regimen remains unclear, several investigators have reported the efficacy of the combination of CDDP and VNR with concurrent radiotherapy for locally advanced stage III NSCLC.^[25–28]^ The median survival times in these studies were approximately 17 to 21 months. These results suggest that concurrent CRT with CDDP and VNR should be considered a standard regimen for the treatment of locally advanced NSCLC. In clinical practice at our institute, the combination of CDDP and VNR is the chemotherapeutic regimen that is most commonly used with concurrent radiotherapy for the treatment of locally advanced NSCLC. The same treatment is often selected for the treatment of postoperative locoregional recurrent NSCLC with curative intent. Our results were in no way inferior to those for the treatment of locally advanced stage III NSCLC reported in previous studies.^[25–28]^ A review of the medical literature produced 2 small series that evaluated the efficacy of concurrent CRT for patients with postoperative recurrent NSCLC. Our results were somewhat superior to those observed in the previous 2 studies (Table 5).^[12,22]^ Four possible explanations for this difference are there. First, more selective patients, all of whom were eligible to receive CDDP-based chemotherapy, were the subjects of this research: the majority of patients in our cohort had a PS of 0 and were younger than 70 years. Second, all the patients received radiotherapy at a dose of ≥60 Gy in the current study. Meanwhile, in previous studies, some of the patients had received low-dose radiation of less than 60 Gy. Thirdly, patients with distant metastases as the initial recurrence after surgery were not included in this study. In contrast, a few cases with distant recurrences were included in previous studies. Fourthly, about 70% of the patients underwent a PET examination at the time of recurrence in the present study, whereas only 6% of patients underwent a similar examination in a previous study.^[22]^ Focusing on the chemotherapeutic regimen, several types of regimens including carboplatin-based chemotherapy were used in the previous studies. Altogether, a more uniform treatment was given to a more uniform group of patients in the present study. Our investigation was well-designed, leading to an accurate portrayal of the treatment outcome of concurrent CRT in the setting of locoregional recurrence, and this result could be worthy of comparison to that for locally advanced stage III disease treated with concurrent CRT.

**Table 5:**
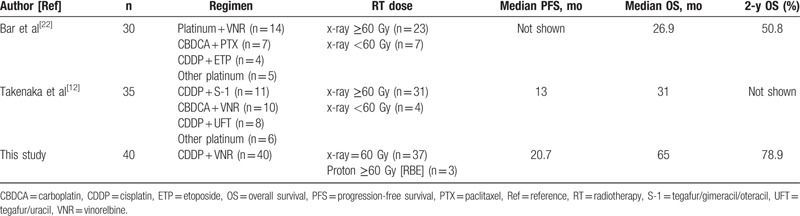
Comparison with previous reports on concurrent chemoradiotherapy for patients with postoperative recurrent nonsmall cell lung cancer.

Regarding the toxicity profile, concurrent CRT with CDDP and VNR was well tolerated. Previous studies in patients with locally advanced stage III NSCLC treated with concurrent CRT with CDDP and VNR have shown that the rates of grade ≥3 leukocytopenia, grade ≥3 neutropenia, grade ≥3 febrile neutropenia, grade ≥3 anemia, grade ≥3 thrombocytopenia, and grade ≥3 esophagitis were in the ranges of 27% to 68%, 19% to 65%, 8% to 14%, 12% to 23%, 1% to 6%, and 4% to 25%, respectively.^[25,27,28]^ The rate of grade ≥3 hematological toxicities without febrile neutropenia in the present study seemed to be lower than the rates of previous studies. Similarly, the rate of grade ≥3 esophagitis in this study was equivalent to or lower than the rates in previous studies. In general, the distribution of disease is expected to be smaller in the setting of locoregional recurrent NSCLC than in the setting of locally advanced stage III disease, which might have led to the use of a smaller radiation field and, consequently, fewer toxicities.

In our cohort, we found that OS was significantly associated with sex in univariate and multivariate analyses. This finding might not have any biological implications. Regarding the patient characteristics, only 1 female patient had squamous cell carcinoma (SCC) and only 1 female patient had rN0-N1 disease. Patients with SCC and patients with rN0-N1 disease tended to have a more favorable prognosis according to the univariate and multivariate analyses. These outcomes might explain why the female patients had an unfavorable prognosis. In addition, the treatment outcomes of female patients with recurrences after CRT might have been poorer than expected. Conversely, factors such as age, pathological stage, surgical procedure, adjuvant chemotherapy, and recurrence-free interval were not identified as prognostic factors for survival in our cohort. In other words, this treatment should be given to all candidates.

The limitations of this study were as follows: first, our analysis was based on a retrospective review performed at a single institution. However, the prevalence of postoperative locoregional recurrent NSCLC is so low that prospective randomized controlled trials are difficult to conduct. Second, a bias in patient selection may be present. Two patients treated with concurrent CRT with carboplatin and paclitaxel were excluded from the current study, but the aim of the present research was to elucidate the efficacy and tolerability of concurrent CRT with CDDP and VNR in patients with postoperative locoregional recurrent NSCLC. When 42 patients were analyzed, including the 2 above-mentioned patients, the treatment outcome was almost the same as that in the presently reported cohort (data not shown). Third, the follow-up time might not have been sufficient, and a certain number of censored cases existed. Further well-designed investigations of this treatment for postoperative locoregional recurrent NSCLC are warranted in the future.

In conclusion, our study revealed that concurrent CRT with CDDP and VNR was active and safe for patients with postoperative locoregional recurrent NSCLC. The outcome of concurrent CRT in the setting of locoregional recurrent disease was not inferior to that in the setting of de novo stage III disease. Salvage CRT for postoperative locoregional recurrent NSCLC might be a promising treatment option for selected patients.
